# Designing of an efficient DC-inducing multi-epitope vaccine against Epstein Barr virus targeting the GP350 using immunoinformatics and molecular dynamic simulation

**DOI:** 10.1016/j.bbrep.2025.101966

**Published:** 2025-03-03

**Authors:** Golzar Fatahi, Maasoume Abdollahi, Zahra Nashtahosseini, Shima Minoo, Mehrnaz Mostafavi, Kholoud Saeidi

**Affiliations:** a Department of Bacteriology, Faculty of Medical Sciences, Tarbiat Modares University, Tehran, Iran; b Department of Anatomical Sciences, Medical Sciences Faculty, Tarbiat Modares University, Tehran, Iran; c Department of Biology, University of Guilan, Rasht, Iran; d Department of Dentistry, Khorasgan Branch, Islamic Azad University, Isfahan, Iran; e Department of Physics, Faculty of Allied Medicine, Shahid Beheshti University of Medical Science, Tehran, Iran; f Shiraz University of Medical Sciences, Shiraz, Iran

**Keywords:** Multi-epitope vaccine, Molecular dynamic simulation, Epstein barr virus, Immunoinformatics

## Abstract

The findings underscore the critical role of Epstein-Barr virus (EBV) in the onset of various cancers. In response to the lack of effective treatments or vaccines for EBV infection, this investigation employed immunoinformatics approaches to develop a potent vaccine targeting multiple epitopes of the EBV glycoprotein 350 (Gp350), a key surface protein. Utilizing computational methods, we designed a comprehensive multi-epitope vaccine featuring 11 CTL and HTL epitopes, totaling 324 amino acids and covering five distinct EBV strains such as B95-8, P3HR-1, GD1, AG876, and Akata. To enhance immunogenicity, the 50S ribosomal protein L7/L12 (rplL) was included as an adjuvant at the vaccine's N-terminal. The vaccine was evaluated for its physicochemical and immunological properties, demonstrating stability, potency, solubility, hydrophilicity, non-allergenicity, and non-toxicity. Molecular docking studies have shown that the vaccine interacts with Toll-like receptor 4 (TLR4). Simulations performed using GROMACS confirmed the stability of the system over 100ns. Immune simulations indicated that the vaccine elicited robust humoral and cellular responses, activating both innate and adaptive immunity. The findings indicate that the multi-epitope vaccine is highly immunogenic and shows significant potential for further experimental validation.

## Highlight

• The molecular dynamic simulation proved the interactions between vaccine and TLR4.
• The new generation vaccine can cover 5 strains such as B95-8, P3HR-1, GD1, AG876, and Akata.
• The integration of immunoinformatics and molecular dynamics simulations has the potential to significantly expedite the vaccine development process.

## 1 Introduction

There are nine different kinds of herpesviruses that may infect humans, and Epstein Barr virus (EBV), also known as Human Gammaherpesvirus 4, is one of them. This type of virus is common to almost every individual. This virus is a DNA virus and contains double-stranded DNA [1]. The size of EBV's genome is approximately 172 kb, and it was the first exemplarily articulated human oncogenic virus [2,3]. Typically, the EBV particle exhibits a tripartite structure: an outer lipid envelope embedded with numerous glycoproteins that facilitate the interaction between the virion and the host cell, a central tegument layer comprising 20–40 proteins, and an internal pseudo-icosahedral nucleocapsid that securely encloses the DNA genome. Previously, detailed atomic models of the EBV icosahedral capsid, the dodecameric portal, and the capsid-associated tegument complex have been established [4,5]. EBV has the ability to infect over 95 % of the global human population and establish a long-lasting infection in the host that persists throughout their lifetime [6]. EBV is distinguished by its ability to infect a variety of cell types, including macrophages, B cells, epithelial cells (EP), natural killer cells (NK), or T-cells [7]. In vitro studies show that EBV infection results in lytic infection in epithelial cells and latent infection in B cells. This suggests that even though these cell types are infected by the same viral strain, their infection patterns differ. Furthermore, EBV enters B cells and epithelial cells by different ways. The involvement of the fusion-triggering proteins gH/gL and gB highlights their critical functions in the process of viral entry [8]. The EBV life cycle is complex and involves the expression of about 80 viral proteins, attesting to the complexity of this viral infection [8,9]. Importantly, only nine proteins have been identified as involved in the transformation of B cells and the induction of tumorigenesis during latent EBV infection. Moreover, nine additional viral proteins are deemed essential for the advancement of EBV infection. These comprise three latent membrane proteins (LMP-1, LMP-2A, and LMP-2B) and six EBV nuclear antigens (EBNA-1, EBNA-2, EBNA-3A, EBNA-3B, -EBNA3C, and EBNA-LP) [10,11]. Although extensive research has been conducted, the exact mechanism underlying the latent-lytic switch remains elusive, necessitating further research [10,11]. Many proteins are created as a result of lytic infection, and these may be divided into three groups according to how they express themselves during various phases of viral replication [12]. The EBV lytic cycle is started by the immediate-early proteins Rta (BRLF-1) and Zta (BZLF-1). The production of late proteins and the replication of the viral genome depend on these immediate-early and early proteins. Although substantial research has been conducted on these lytic gene products, many of their functions remain incompletely understood [12]. Glycoprotein 350 (gp350) of EBV is identified as one of the most prevalent glycoproteins on the virus envelope, serving as the main target for neutralizing antibodies and crucial for the virus's attachment to B lymphocytes. Many studies have investigated EBV gp350 as a potential vaccine target, and this study focused specifically on it [13]. EBV, the first oncogenic virus identified, is notable for its capacity to establish a persistent infection in humans. EBV is not only responsible for infectious mononucleosis but is also closely associated with several cancers. In a serious attempt to combat EBV infection, several vaccine formulations have undergone extensive testing in a variety of animal models and human studies. However, despite these comprehensive trials, no vaccine has been approved for preventing EBV infection [14]. It is a troubling reality that the scientific community still faces significant challenges in effectively combating this persistent viral pathogen [9]. The development of the vaccine is important that one of the useful way is immunoinformatics approaches that are functional against different viruses such as SARS-CoV-2, MERS-CoV, influenza [[15], [16], [17]], and others. In this creative investigation, the utilization of immunoinformatics and structural analysis aid in facilitating the development of a highly effective vaccine characterized by its encompassing nature and high degree of conservancy, thereby accounting for both types of EBV. Additionally, the comprehensive analysis of epitopes and construction of multi-epitope has yielded to validate the structural analysis that the efficacy of the vaccine increased through inserting the adjuvant and other sequences.

## 2 Method and material

### 2.1 Retrieval of protein sequence and analysis of target

**Fig. 1:**
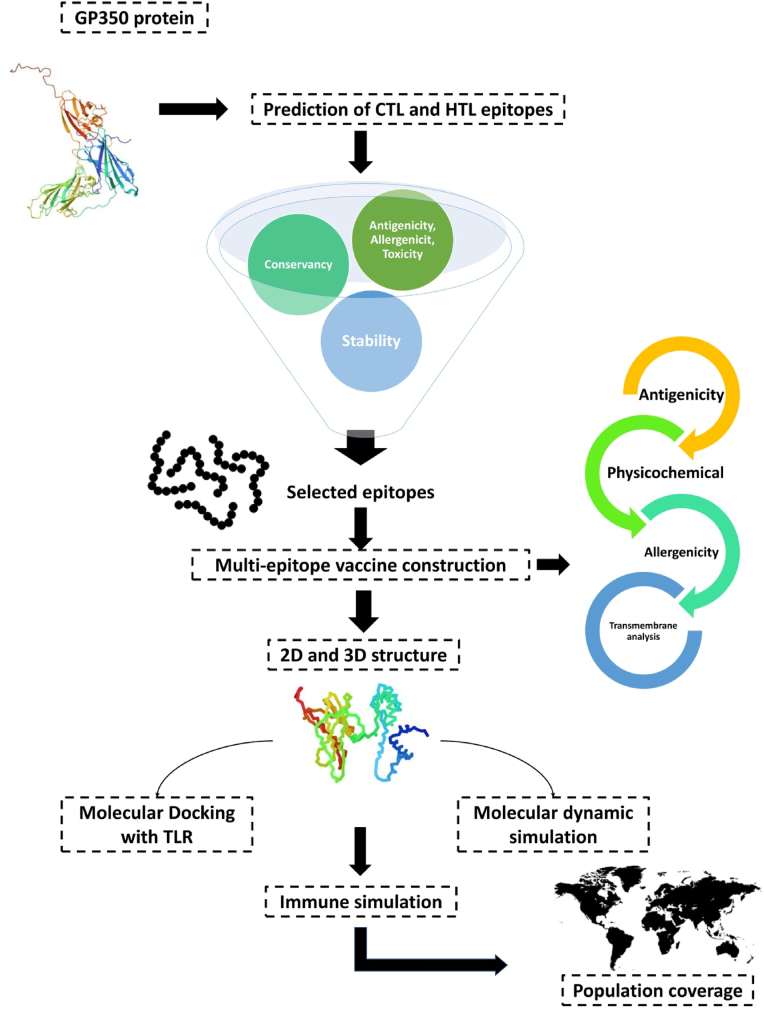
The pathway of designing the potent multi-epitope vaccine.

In this important part of the study, we have successfully obtained the glycoprotein 350 (GP350) from NCBI. Our investigation focused on two different types of EBV that contained type 1 (Type A) and type 2 (Type B) [18]. To accomplish this, we carefully selected a range of strains including B95-8, P3HR-1, GD1, AG876, and Akata. Moreover, these strains were subjected to an analysis of their protein sequences using the MEGA11 tool [19]. This software enabled us to align the sequences using the Multiple Sequence Comparison by Log-Expectation (MUSCLE) method, facilitating the identification of any differences or mutations between the strains. Also, our goal is to utilize this information to design a comprehensive vaccine that effectively covers all the variants of EBV. The workflow of the investigation is demonstrated in Fig. 1.

### 2.2 T-cell epitopes prediction

In vaccine development, the presentation of peptides by major histocompatibility complex I (MHC-I) is crucial for activating the immune system, making it essential for selecting CTL epitopes. We used the default prediction method of the IEDB tool (http://tools.iedb.org/mhci/) to forecast how MHC-I peptides bind to frequently encountered human alleles. We used NetMHCpan 4.1 EL to predict CTL epitopes, and it worked well for this. MHC-II molecules activate CD4 T-lymphocytes, which is a critical component of the adaptive immune response. To find and choose MHC-II peptide binders from the listed antigens, we used the IEDB MHC-II epitope prediction tool, which is a useful resource. We selected the IEDB-recommended epitope prediction methods over others, even though there were other options available, because of their track record of accuracy and dependability in prior applications.

### 2.3 Evaluation of epitopes

Several critical factors must be considered when selecting the optimal epitopes. One key aspect is identifying epitopes with high antigenicity, which means they can elicit a strong immune response, and high immunogenicity, indicating their capability to stimulate an immune reaction. It is also essential to ensure that the chosen epitopes are non-allergenic and non-toxic to avoid any adverse effects in the host. Additionally, epitopes that are soluble and stable are preferred, as these properties enhance their effectiveness. Finally, epitopes with high conservancy, meaning they are preserved across various EBV strains or variants, are crucial. One concern in immunization is the potential for increased immune system reactivity to vaccine components. To mitigate this issue, the AlgPred and AllerTop server (http://crdd.osdd.net/raghava/algpred/) provides a useful tool for predicting protein allergenicity. This prediction is based on similarities between the protein and known IgE epitopes. Additionally, VaxiJen v2.0 (http://www.ddg-harmfac.net/vaxijen) uses an alignment-free method to predict antigenicity by evaluating the physicochemical properties of proteins. To assess epitope toxicity, we utilized the ToxinPred tool. [20]. On the other hand, we used the conservancy tool from IEDB to predict highly conserved epitopes. These highly conserved epitopes were chosen along with other optimal conditions discussed earlier. For the subsequent stability analysis, we employed the Protparam server (https://web.expasy.org/protparam/). Finally, to prevent autoimmune reactions, we used Blastp to ensure that the selected peptides do not have similarities with human proteins.

### 2.4 Vaccine design

First, it's crucial to remember that the stiff EAAAK linker was used to splice the 50S ribosomal protein L7/L12 at the vaccine's N-terminal. Research is now being done to determine if the 50S ribosomal protein L7/L12 (rplL) used as an immunoadjuvant in tumor immunotherapy involving dendritic cells (DCs), especially if it can cause DC maturation [21]. This strategic insertion of the adjuvant was implemented with the primary objective of augmenting the immunogenicity of the vaccine. Moreover, following the aforementioned linker, the pan-HLA DR binding epitopes, specifically the PADRE epitope 13aa, were meticulously incorporated into the vaccine [22]. The purpose of the addition was to improve the immunogenicity of the vaccine. In order to achieve an extended conformation, which enhances flexibility, helps with appropriate protein folding, and makes functional domain separation easier, linkers are necessary. In order to create a more strong and stable protein structure, several variables are essential. To successfully incorporate CTL epitopes, also known as MHC-I binders, a suitable AAY linker was utilized [23,24]. Conversely, the HTL epitopes were joined using the GPGPG linker, which demonstrated high effectiveness for this purpose. To accurately classify the epitope types, a GGGS linker was utilized [25]. Lastly, the vaccine's C-terminal was modified to include TAT motif (11 amino acids). The purpose of this exact alteration was to guarantee that the vaccination had the desired characteristics and abilities. These meticulous adjustments were strategically implemented to enhance the vaccine's effectiveness and improve its immunogenicity.

### 2.5 Assessment of physicochemical parameters, allergenicity, and antigenicity of selected vaccine

The allergenicity of the multi-epitope vaccination was predicted using the AllerTOP v.2.0 server. [26]. The antigenicity of the designed vaccines was assessed using the VexiJen v2.0 server [27], with a threshold value of 0.5. Additionally, ANTIGENpro (http://scratch.proteomics.ics.uci.edu) was used to assess the antigenicity of the vaccination designs. We made use of the ToxinPred server to assess the toxicity of the created vaccinations. Furthermore, a number of physicochemical properties of the vaccine constructs, including molecular weight, Grand Average of Hydropathicity (GRAVY), theoretical isoelectric point (pI), instability index, aliphatic index, and half-life, were estimated using the Expasy ProtParam tool (https://web.expasy.org/protparam). We used the SOLpro web program (http://scratch.proteomics.ics.uci.edu) to evaluate solubility. To forecast the solubility of a protein, this program use a two-stage support vector machine (SVM) architecture with several representations of the protein's main sequence. Assessing the solubility of proteins that are involved in the creation of vaccines is important since it has a big influence on how well they work. Finally, the number of transmembrane helices in the suggested vaccine designs was predicted using the TMHMM [28].

### 2.6 Prediction of 2D structure

To accurately forecast the distribution of helices (H), strands (S), and coils (C) in the secondary structure, we utilized the PSIPRED and SOPMA servers (http://expasy.org/tool/gor4.html and https://npsa.lyon.inserm.fr/cgi-bin/npsa_automat.pl?page=/NPSA/npsa_sopma.html).

### 2.7 3D structure prediction, refinement, and validation of multi-epitope vaccine

The three-dimensional (3D) structure is another name for the tertiary structure. The AlphaFold2 algorithm was utilized to anticipate the EBV vaccine's three-dimensional structure [29]. The three-dimensional protein model obtained from the trRosetta server was enhanced using the GalaxyRefine web servers (http://galaxy.seoklab.org/cgi-bin/submit.cgi?type=REFINE) [30]. The general validity of the model has been confirmed by the SAVES server v6.0 (https://saves.mbi.ucla.edu/) and the ProSA-web server (https://prosa.services.came.sbg.ac.at/prosa.php) [31]. The optimum model for the structure of the multi-epitope vaccine was determined by analyzing the ERRAT and Ramachandran plot.

### 2.8 B-cell epitopes prediction

Since B-cell epitopes are essential for immune system activation, they are also essential for antigen identification and their interaction with antibodies. Accurately pinpointing the location of B-cell epitopes is crucial to the advancement of vaccination science. Protein 3D structures were analyzed to predict discontinuous and linear B-cell epitopes using the ElliPro service (http://tools.iedb.org/ellipro/). This tool employs a scoring method known as the Protrusion Index (pI) to estimate the likelihood of epitope presence. By evaluating pI values, it is possible to identify discontinuous epitopes, which are clustered based on their spatial proximity as measured by distance R (Å). This approach facilitates the discovery and characterization of B-cell epitopes and provides insightful information for the creation of potent vaccinations [32,33].

### 2.9 Molecular docking

Using the ClusPro2.0 website (https://cluspro.org/help.php), we performed ligand-receptor docking analysis to evaluate the interaction between the vaccinations and the human Toll-like receptor-4 (PDB: 3FXI) [34]. The server defaults to displaying only the top 10 models. For visual analysis, Chimera software was utilized to assess protein-protein binding interactions between the vaccine designs and the TLR4-vaccine complex. Additionally, the PDBsum server was employed to analyze protein-protein interactions, focusing on hydrogen bonds (H-bond), ion bridges, and the overall number of interactions to determine the optimal energy configuration of the docked complex [35].

### 2.10 Molecular dynamic simulation

The molecular dynamics simulation (MDS) was employed by GROMACS tools that are designed to simulate protein complexes [36]. To assess the protein structure, stability, and interactions, we used MDS. The 2023 version of GROMACS software was utilized with the OPLS-AA force field and the spc216 water model, incorporating Na+ and Cl-ions at a 0.15 % concentration for neutralization in a cubic box (final dimensions: 13.91 nm × 13.91 nm × 13.91 nm). Following system setup, the vaccine-TLR4 complex's energy minimization (EM) was carried out over 5000 steps utilizing the steepest descent approach [37]. Subsequently, the system underwent the NVT step, where it was gradually heated from 0 K to 310 K using the Berendsen thermostat. Following this, the NPT step was performed to maintain the pressure at 1 atm and facilitate equilibration over 100 ps. Finally, a 100 ns molecular dynamics (MD) simulation was carried out on all prepared complexes, and the trajectory data was extensively analyzed, including assessments of Root Mean Square Deviation (RMSD) and Radius of Gyration (Rg) [37].

### 2.11 Binding energy

The binding free energy (ΔG bind) for the ligand molecules was calculated using the Prime MM-GBSA method, based on the docked pose obtained from the Glide algorithm. The binding mode of the complex structures was visually examined using the ligand interaction diagram tool in Schrodinger to better understand the interaction.

The formula corresponds to the (ΔG bind) is given below:

ΔG(bind)=ΔG(solv)+ΔE(MM)+ΔG(SA)

### 2.12 Immune simulation

The C-ImmSim server was used to do computational immunological simulation in order to ascertain the immune response that the multi-epitope vaccination induced in the host organism [38,39]. To explore the cellular and humoral responses of the mammalian immune system to the formulated vaccine, three doses were administered at intervals of 35 days, consistent with previous studies. The simulation was conducted over 1050 steps, with vaccine administrations occurring at time steps 1, 21, and 45. The remaining simulation parameters were kept at their default settings. This approach enabled a detailed investigation of immune response dynamics and the vaccine's effectiveness. The C-ImmSim server was employed to provide an in-depth analysis of the complex interactions between the immune system and the multi-epitope vaccine, offering insights into the underlying mechanisms of the immune response.

### 2.13 Population coverage

Population coverage of epitopes was determined utilizing the IEDB (http://tools.iedb.org/population/) for the prioritized epitopes. This methodology was employed due to its capability to ascertain the proportion of the population capable of mounting a response to the specific epitope. Furthermore, it aids in understanding the extent to which the population can generate an immune reaction to the epitope.

## 3 Result

### 3.1 T-cell epitopes prediction and selection

The analysis of the gp350 targets facilitated the identification of MHC-I and MHC-II epitopes using the IEDB server. A total of 7 MHC-I and 4 MHC-II epitopes were subsequently selected and evaluated based on antigenicity, stability, and conservancy. Antigenicity scores were obtained through the Vaxijen server, while the Protparam server was used to assess the stability of the epitopes, with only the stable ones being retained. To ensure broad applicability, the epitopes were further analyzed across five different strains of Epstein-Barr Virus (EBV), prioritizing those with high conservancy. The selected epitopes, which were non-allergenic and non-toxic, were then chosen as candidates for the design of an EBV vaccine. Additionally, a BLASTp analysis confirmed that none of the epitopes showed homology to human proteins. The selected epitopes are presented in Table 1, Table 2.

**Table 1:** The predicted CTL epitopes.

MHC I	Alleles	Vaxijen	Score	Conservancy
AEMQNPVYL	HLA-B∗40:01	0.58	0.99	5/5 (100 %)
TSSKLRPRW	HLA-B∗57:01	1.79	0.99	4/5 (80 %)
QPRFSNLSM	HLA-B∗07:02	1.8	0.98	5/5 (100 %)
TPNATSPTL	HLA-B∗07:02	0.41	0.97	5/5 (100 %)
MPTNTTDITY	HLA-B∗35:01	0.94	0.96	5/5 (100 %)
STSSKLRPRW	HLA-B∗57:01	1.60	0.95	4/5 (80 %)
RLTPRPVSR	HLA-A∗31:01	1.99	0.95	5/5 (100 %)

**Table 2:** The predicted HTL epitopes.

MHC II	Alleles	Vaxijen	Score	Instability index	Conservancy
NSTNITAVVRAQGLD	HLA-DQA1∗01:02/DQB1∗06:02	1.07	0.89	Stable	100 %
TAVVRAQGLDVTLPL	HLA-DRB4∗01:01	0.7	0.89	Stable	40 %
QPKNATSAVTTGQHN	HLA-DQA1∗01:02/DQB1∗06:02	0.79	0.73	Stable	40 %
CNSTNITAVVRAQGL	HLA-DQA1∗01:02/DQB1∗06:02	0.73	0.75	Stable	100 %

### 3.2 Vaccine construct

**Fig. 2:**
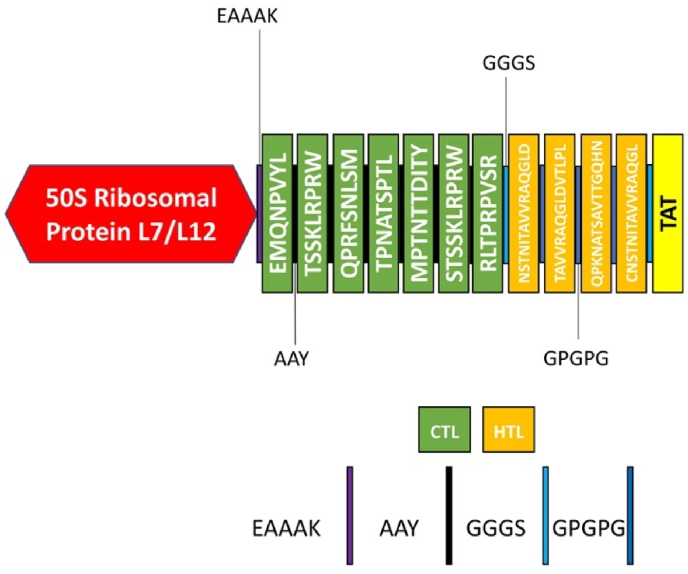
Schematic presentation of the final multi-epitope vaccine against EBV. The 329 amino acid sequence that containing adjuvant at N-terminal, HTL, CTL, and TAT sequence that linked with Linkers.

To create a novel vaccine that satisfies a number of strict requirements, including binding affinity, antigenicity, non-toxicity, and non-allergenicity, the 7 CTL and 4 HTL epitopes were nominated. A novel strategy was used to increase the vaccine's immunogenicity in addition to these important factors. The adjuvant, 50S ribosomal protein L7/L12, was added to the vaccine construct's N-terminal region. The integration of EAAAK linkers was strategically employed to facilitate the attachment of the epitopes, while AAY linkers were specifically utilized to interconnect the CTL epitopes, and the HTL epitopes were intricately linked together using GPGPG linkers. The TAT sequence was carefully added to the C-terminal end of the vaccine construct to improve the design even more. After these meticulous design steps, the vaccine sequence underwent thorough testing to evaluate its antigenicity, non-allergenicity, non-toxicity, solubility, and overall compliance with the predefined criteria. The detailed illustration portraying the final multi-epitope vaccine peptide developed in the present investigation can be found in Fig. 2.

### 3.3 Evaluation of vaccine

Using VaxiJen and ANTIGENpro, the antigenicity of the multi-epitope vaccine design was investigated. Both analyses showed that the vaccine had the potential to be a great resource antigen, with a score of 0.54. Upon analysis of the vaccine sequence on the AllerTOP server, it was determined to be non-allergenic. The molecular weight of the final protein was calculated to be 33.4 kDa, along with a theoretical isoelectric point (pI) score of 8.48. Furthermore, experimental data suggested that the half-life of the protein was approximately 100 h in mammalian reticulocytes in vitro, over 20 h in yeast, and around 10 h in E. coli in vivo. The calculated instability index (II) value of 27.20 indicated that the protein possessed a stable nature, given that an II value exceeding 40 typically signifies instability. Moreover, the high aliphatic index score of 84.71 pointed towards the protein's thermos ability. The Grand average of hydropathicity was determined to be −0.049, highlighting the hydrophilic nature of the vaccine constructs. Additionally, the solubility score of the protein was found to be 0.89, indicating that the protein would exhibit high solubility upon expression (Table 3). At last, there weren't detected transmembrane helices in the vaccine construct.

**Table 3:** The Characterization of the multi-epitope vaccine against EBV.

Number of amino acids	Molecular weight	TMHMM	Antigenicity	Solubility (Solpro)	Half-time (mammalian reticulocytes, in vitro)	Instability index	Aliphatic index	GRAVY
329	33434.01	Non	Vaxijen (0.59), AntigenPro (0.90)	Soluble (0.86)	30h	27.20	84.71	−0.049

### 3.4 Prediction of 2D structure

**Fig. 3:**
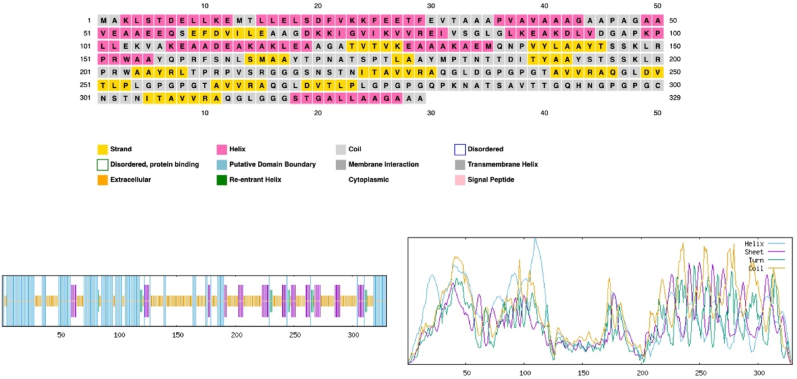
The 2D structure of the vaccine.

The complete vaccine sequence was approximated to consist of 34.95 % α-helix, 15.20 % β-strand, 3.34 % Beta turn, and 46.50 % coil, as illustrated in Fig. 3.

### 3.5 3D model prediction, refinement, and validation

**Fig. 4:**
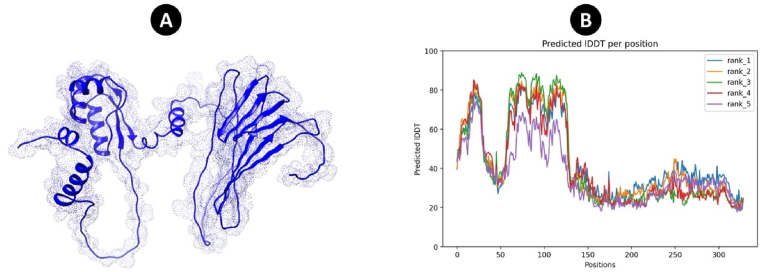
Protein 3D modeling was predicted by AlphaFold. (A) The tertiary structure of multi-epitope vaccine (B) The plot of lDDT (Local Distance Difference Test) per position for prediction of 3D structure.

**Fig. 5:**
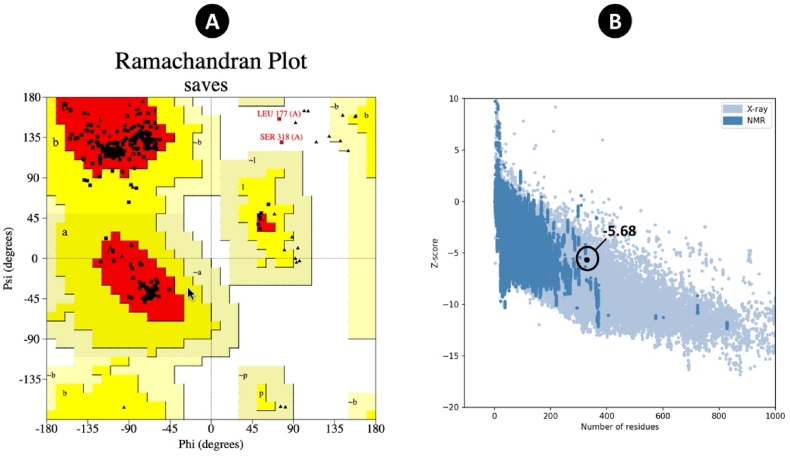
The quality assessment of the final multi-epitope 3D structure. (A) Ramachandran plot (B) The Z-score was evaluated by ProSA-web server.

AlphaFold2 predicted the five models. Next, the most effective multi-epitope vaccination model was chosen for further development (Fig. 4). The refinement employed by Galaxyrefine server presented the 5 models that all of the models were evaluated by different tools such as ERRAT, Ramachandran plot, and Z-score (Fig. 5). PROCHECK, ERRAT, and ProSA-web were utilized to contrast the final quality of the 3D structure of the multi-epitope vaccine before and after refinement (Table 4). The Ramachandran plot of the beginning model showed that 59.66 %, 25.9 %, 9.6 %, and 4.8 % of the residues were present in the favoured, additional allowed, generously allowed, and disallowed regions, respectively. Following refinement, the percentage of residues in the preferred, additional allowed, generously allowed, and forbidden areas were 95.6 %, 3.7 %, 0 %, and 0.7 %, respectively. According to the ERRAT, the ultimate quality factor for the original model would be 63.30, and for the enhanced model, it would be 77.24. Following refining, the Z-score of the original model, which was found to be −5.27, increased to −5.68.

**Table 4:** The assessment of the 3D structure of multi-epitope vaccine.

	Ramachandran plot				ERRAT	ProSA-web
	Most Favoured region	Additional allowed regions	Generously allowed regions	Disallowed regions		Z-score
Model 1	59.6%→95.6 %	25.9%→3.7 %	9.6%→0%	4.8%→0.7 %	63.30→77.2	−5.27→5.68

### 3.6 B-cell epitopes prediction

**Fig. 6:**
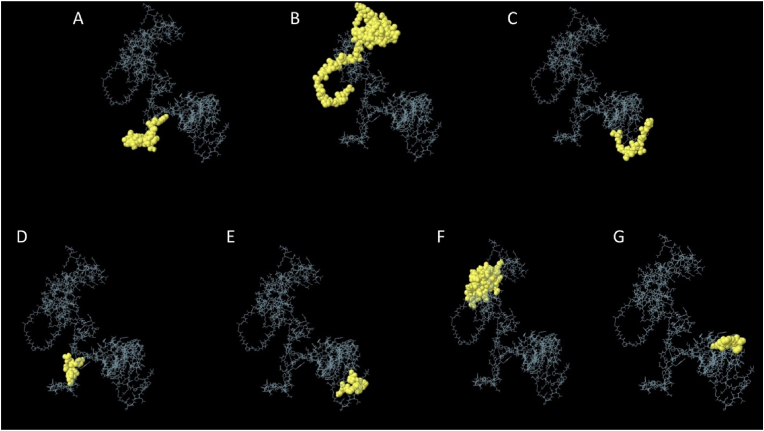
The 3D representation conformational B-cell epitopes of constructed multi-epitope vaccine. (A) 20 residues with 0.84 score (B) 51 residues with 0.83 score (C) 17 residues with 0.71 score (D) 6 residues with 0.69 score (E) 8 residues with 0.65 score (F) 36 residues with 0.64 score (G) 9 residues with 0.62 score.

The ElliPro tool identified 7 conformational B-cell epitopes and 11 linear B-cell epitopes. The linear epitopes ranged from 4 to 52 amino acids in length, while the conformational epitopes spanned from 9 to 51 amino acids (Table 5). The conformational epitopes are shown in (Fig. 6). The vaccination adjuvant was shown to have the highest scores (0.84, 0.83) for both conformational and linear epitopes, indicating its critical function in inducing humoral response.

**Table 5:** The conformational epitopes that predicted via Ellipro server.

No.	Residues	Number of residues	Score
1	A:S161, A:N162, A:L163, A:S164, A:M165, A:A166, A:A167, A:Y168, A:T169, A:P170, A:N171, A:A172, A:T173, A:S174, A:P175, A:T176, A:L177, A:A178, A:A179, A:Y180	20	0.848
2	A:M1, A:A2, A:K3, A:L4, A:S5, A:T6, A:D7, A:E8, A:L9, A:L10, A:K11, A:E12, A:M13, A:T14, A:L15, A:L16, A:E17, A:L18, A:S19, A:D20, A:F21, A:V22, A:K23, A:K24, A:F25, A:E26, A:E27, A:T28, A:F29, A:V31, A:T32, A:A33, A:A34, A:A35, A:P36, A:V37, A:A38, A:V39, A:A40, A:A41, A:A42, A:G43, A:A44, A:A45, A:P46, A:A47, A:G48, A:A49, A:A50, A:V51, A:E52	51	0.832
3	A:G313, A:L314, A:G315, A:G316, A:G317, A:S318, A:T319, A:G320, A:A321, A:L322, A:L323, A:A324, A:A325, A:G326, A:A327, A:A328, A:A329	17	0.719
4	A:A154, A:A155, A:Y156, A:Q157, A:P158, A:F160	6	0.679
5	A:G275, A:P276, A:G277, A:P278, A:G279, A:Q280, A:P281, A:K282	8	0.659
6	A:A66, A:A67, A:G68, A:D69, A:K70, A:K71, A:I72, A:G73, A:V74, A:I75, A:K76, A:V77, A:R79, A:E80, A:V82, A:S83, A:G84, A:L85, A:G86, A:L87, A:K88, A:E89, A:A90, A:K91, A:D92, A:V94, A:D95, A:G96, A:A115, A:K116, A:L117, A:E118, A:A119, A:A120, A:G121, A:A122	36	0.648
7	A:H293, A:N294, A:G295, A:P296, A:G297, A:P298, A:G299, A:C300, A:N301	9	0.629

### 3.7 Molecular docking

**Fig. 7:**
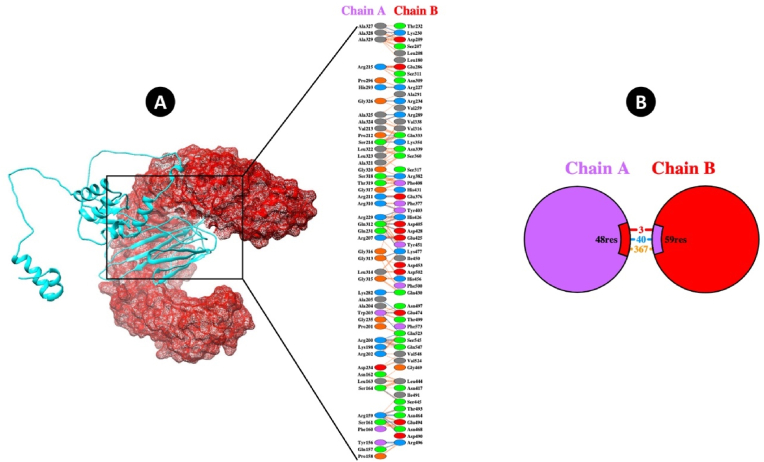
Molecular docking and protein-protein interaction analysis. (A) The TLR4 and multi-epitope vaccine with their interaction that consist of H-bond, ion bridge. (B) The other visualization of protein-protein interaction.

Using the ClusPro program, which predicted the 29 poses, molecular docking between the multi-epitope vaccination and TLR4 was implemented. The optimal position was chosen for molecular dynamic simulation because, in comparison to other positions, its energy was negative (−1419.2 kcal/mol). The analysis of the protein-protein interaction carried out by the PDBsum tool that shown in Fig. 7. There is interaction between the 59 residues of chain A (Vaccine) and the 48 residues of chain B (TLR4). 40 hydrogen bonds and the three ion bridge were anticipated. The amino acids that formed these H-bond showed in Table 6.

**Table 6:** The protein-protein interaction between Vaccine: Chain A and TLR4: Chain B.

Hydrogen Bonds							
No.	Atom 1	Residue 1	Chain 1	Atom 2	Residue 2	Chain 2	Distance (Å)
1	NE2	HIS 293	A	NE	ARG 227	B	3.08
2	O	ALA 327	A	NZ	LYS 230	B	2.61
3	O	ALA 328	A	NZ	LYS 230	B	2.60
4	O	ALA 329	A	NZ	LYS 230	B	2.73
5	O	GLY 326	A	NH1	ARG 234	B	2.67
6	O	ALA 324	A	NH1	ARG 289	B	2.56
7	O	ALA 325	A	NH1	ARG 289	B	2.75
8	N	SER 214	A	OE1	GLN 333	B	2.69
9	O	PRO 212	A	NE2	GLN 333	B	2.95
10	OG	SER 214	A	NZ	LYS 354	B	2.66
11	NE	ARG 211	A	OE1	GLU 376	B	2.84
12	O	SER 318	A	NE	ARG 382	B	3.00
13	O	SER 318	A	NH2	ARG 382	B	2.92
14	NH1	ARG 229	A	OH	TYR 403	B	3.04
15	NH1	ARG 310	A	OH	TYR 403	B	2.65
16	NE2	GLN 312	A	OD2	ASP 405	B	2.96
17	NH2	ARG 207	A	OE1	GLU 425	B	2.69
18	NH2	ARG 229	A	NE2	HIS 426	B	3.02
19	OE1	GLN 312	A	NE2	HIS 426	B	3.03
20	NE2	GLN 312	A	OD2	ASP 428	B	2.86
21	NZ	LYS 282	A	OE1	GLN 430	B	2.69
22	OG	SER 164	A	OG	SER 445	B	2.77
23	NH1	ARG 159	A	O	ASN 464	B	3.31
24	NH2	ARG 159	A	O	ASN 464	B	2.72
25	NH1	ARG 159	A	OD1	ASN 468	B	2.68
26	N	ALA 205	A	OE1	GLU 474	B	2.89
27	NE1	TRP 203	A	OE2	GLU 474	B	2.89
28	O	GLN 312	A	NZ	LYS 477	B	2.74
29	O	GLY 313	A	NZ	LYS 477	B	2.65
30	NH2	ARG 159	A	O	ASP 490	B	2.77
31	NE	ARG 159	A	OG1	THR 493	B	2.89
32	N	SER 161	A	OE2	GLU 494	B	2.99
33	O	TYR 156	A	NE	ARG 496	B	2.74
34	O	TYR 156	A	NH2	ARG 496	B	2.79
35	O	GLN 157	A	NH2	ARG 496	B	2.63
36	N	TRP 203	A	OE1	GLN 523	B	3.09
37	O	ARG 200	A	NE2	GLN 523	B	2.98
38	NH1	ARG 200	A	O	SER 545	B	2.97
39	NH2	ARG 200	A	OG	SER 545	B	2.74
40	NH1	ARG 200	A	OE1	GLN 547	B	2.71

Salts bridges							
No.	Atom 1	Residue 1	Chain 1	Atom 2	Residue 2	Chain 2	Distance (Å)
1	NH2	ARG 215	A	OE2	GLU 286	B	2.77
2	NH2	ARG 211	A	OE1	GLU 376	B	2.79
3	NH2	ARG 207	A	OE1	GLU 425	B	2.69

### 3.8 Molecular dynamic simulation

**Fig. 8:**
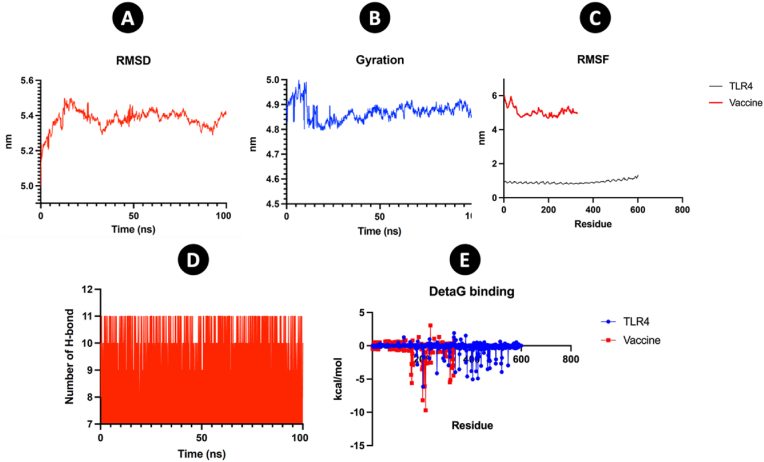
Molecular dynamic simulation. The analyzed were employed by C-alpha (A) Root mean square deviation (RMSD) (B) Gyration (C) RMSF (D) H-bond (E) DetaG.

GROMACS 2019 tools utilized MDS to evaluate the stability of the interaction between TLR4 and the multi-epitope vaccination. The equilibration of 2 phases were carried out in 310 k and the MD run was employed to measure the stability of the EBV multi-epitope vaccine. The RMSD profile of the complex generated during the MD simulation in 100ns shows structural stability. The RMSD value of the complex increased to 5.5 nm and then decreased to 5.3 nm during 30ns and after that got stable between 5.35 nm and 5.45 nm in 100ns (Fig. 8A). The observed high RMSD values indicate a significant degree of flexibility in the vaccine structure, which can be attributed to the presence of coil regions within its composition. Coils are inherently flexible and dynamic structural elements, lacking the stable hydrogen bonding networks. This intrinsic flexibility allows the vaccine construct to adopt multiple conformations during molecular dynamics simulations, which is often essential for efficient interaction with immune receptors, such as TLRs, enhancing antigen presentation and immune activation. Such flexibility can also contribute to the vaccine's ability to adapt to various conformational states required for optimal epitope display. The other side of the analysis of Rg showed the stiffness of a vaccine construct. The initial of MD simulation demonstrated the increase until 10ns after that between 10ns and 20ns decreased and after that got stable (Fig. 8B). The analysis of RMSF provided valuable insights into the dynamic behavior of the vaccine structure, particularly highlighting changes in regions dominated by coils. Coils, being non-regular secondary structures, inherently exhibit significant flexibility due to the lack of stable hydrogen bonds and ordered conformation seen in α-helices and β-sheets. This flexibility enables the vaccine to undergo dynamic structural rearrangements, which may facilitate its adaptability for effective immune receptor binding. In contrast, the analysis of TLR4 revealed a stable condition, primarily attributed to its β-sheet-dominant structure. β-sheets are more rigid and stable due to the presence of strong inter-strand hydrogen bonding, which minimizes conformational fluctuations. However, the regions of high RMSF observed in TLR4 were specifically localized to coil regions, underscoring the inherent flexibility of these non-structured regions. This balance of flexibility in coils and stability in β-sheets reflects the dynamic yet robust interaction interface required for the vaccine-TLR4 complex to perform its immune-stimulating role efficiently [40,41] (Fig. 8C).

The analysis of hydrogen bonds between the vaccine and TLR4 over a 100 ns molecular dynamics simulation reveals significant and stable interactions that play a crucial role in the stability of the complex. The number of hydrogen bonds fluctuates within a narrow range, predominantly between 8 and 11 throughout the 100 ns simulation. The lower bound remains consistent around 8 hydrogen bonds, while the upper limit occasionally reaches 11 bonds. This trend indicates that the interaction remains stable without significant disruptions during the simulation period. The relatively stable nature of the hydrogen bonds suggests a robust and consistent interaction between the vaccine construct and TLR4. The absence of sharp drops or dramatic increases in bond numbers indicates that the binding interface remains largely intact throughout the simulation. This stability is critical, as it reflects the vaccine's ability to persistently interact with TLR4, which is a key immune receptor (Fig. 8D).

### 3.9 MMGBSA

The total binding energy of −195.15 kcal/mol suggests a stable interaction between the vaccine candidate and TLR4. The VDE value of −261.79 kcal/mol indicates favorable van der Waals interactions, which contribute to the compactness and tight binding of the complex (Table 7). van der Waals interactions are crucial for stabilizing the molecular recognition between the TLR4 receptor and the vaccine, ensuring that the latter can efficiently activate immune responses. The electrostatic energy (ELE) of −1639.25 kcal/mol is significantly negative, highlighting strong electrostatic attraction between the negatively charged components of the vaccine and the positively charged residues on TLR4. This attraction is essential for the formation of a stable TLR4-ligand complex and can enhance the receptor's ability to recognize and respond to the vaccine, facilitating immune activation. The Generalized Born energy (GB) of 1741.14 kcal/mol suggests that the solvent environment favors the binding, but the positive value indicates some opposition to the binding due to solvent effects. This is a common feature in protein-ligand interactions, where the polar and nonpolar solvent environments can influence the stability of the complex. The balance of this energy with other components (like van der Waals and electrostatic energies) indicates a favorable binding despite the solvent's influence. The Solvent Accessible Surface Area (SA) value of −35.25 kcal/mol further suggests favorable hydration effects, as a negative value typically indicates that the surface area exposed to solvent is minimized upon complex formation, which often leads to a more stable complex in aqueous environments. In summary, the negative total energy indicates that the vaccine-TLR4 complex is energetically stable, with significant contributions from favorable van der Waals and electrostatic interactions. This stability suggests that the vaccine candidate may effectively bind to TLR4, triggering the desired immune responses. The energy values support the potential of the vaccine in engaging TLR4 to initiate signaling pathways crucial for immune activation, thus providing a solid foundation for its development as an effective immunogen.

**Table 7:** The MMGBSA result.

TLR4-Vaccine	
Energy	Value (kcal/mol)
**VDE**	−261.79
**ELE**	−1639.25
**GB**	1741.14
**SA**	−35.25
**TOTAL**	−195.15

### 3.10 Computational immune

**Fig. 9:**
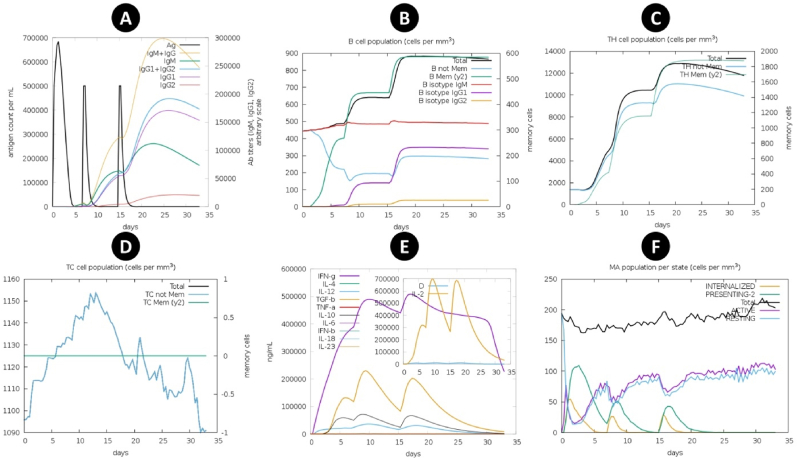
Computational immune simulation. (A) Antibodies production and vaccine injection (B) B-cell population (C) T-helper cell population (D) T-cytotoxic cell population (E) Cytokines production (F) Macrophage population.

The C-ImmSim program was utilized to assess the immunological response to the multi-epitope vaccination after 35 days. The initial injections led to an increase in antigen levels, while the second or booster injections resulted in a decrease. As shown in Fig. 9A, the level of IgM and IgG increased following the primary and booster injections. Following the second and booster injections, a modest population of B-cell isotype IgG2 was detected, along with the memory and isotype IgM and IgG1 populations of the B-cells following each injection (Fig. 9B). The Th cell population reached to 5000 cells per mm^3^ and after the second and booster injections increased to 10000 and 13000 mm^3^ during 35 days (Fig. 9C). Furthermore, following each injection, there was a notable rise in Th memory cells, with the biggest increase following the booster dose. The Tc reached 1150 mm^3^ on 14th day after the second injection (Fig. 9D). Each injection led to a significant rise in cytokines, including IFN-Gamma (reaching 500,000 ng/mL), IL-2, and TGF-b levels (Fig. 9E). The macrophage population became activated following multiple injections (Fig. 9F).

### 3.11 Population coverage

**Fig. 10:**
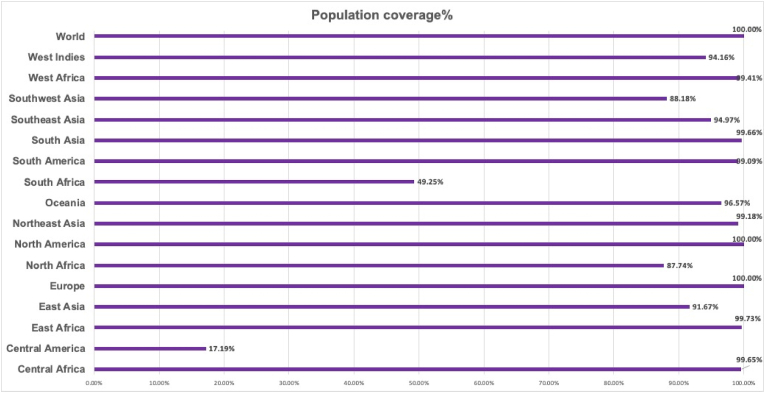
Population coverage. Most of the population of the world have significant coverage.

For the 11 CTL and HTL epitopes that were chosen, the worldwide coverage was assessed. Strongly binding MHC epitope-containing alleles were taken into consideration in the population coverage analysis. The chosen gp350 protein epitopes showed a total coverage of 100 % worldwide, with Europe having the highest coverage at 100 %, and South Africa showing the lowest coverage at 49.25 %. The other coverage is shown in Fig. 10.

## 4 Discussion

Vaccination is one of the most effective ways to guard against harmful diseases. Different types of vaccines, such as peptide, subunit, DNA, and mRNA vaccines, were used to trigger an immune response. In this case, the use of immunoinformatics techniques allowed researchers to analyze all the proteins in the virus to develop the most effective EBV vaccine. However, the spread of EBV could lead to significant issues in the future, and the powerful vaccine has not been approved to prevent EBV infection [42]. Numerous pieces of evidence connect gastrointestinal and nasopharyngeal cancers to EBV. Certain studies offer compelling evidence that EBV contributes to the pathophysiology of breast cancer [[43], [44], [45], [46]]. The significance of EBV was emphasized through the development of an effective multi-epitope vaccine. In this study, we used in-silico analysis to design a potent peptide-based vaccine against EBV, acknowledging the advantages of multi-epitope vaccines in producing specific immune responses. The vaccine was composed of four MHC-I and seven MHC-II epitopes, which were designed to trigger humoral, cellular, and innate immune responses. We employed the IEDB server to predict potent epitopes targeting GP350. Based on their stability and ability to cover a variety of EBV strains, such as B95-8, P3HR-1, GD1, AG876, and Akata, the selected epitopes were made. Additionally, antigenicity, physicochemical properties, and conservancy were considered during the selection process.

The MHC class I epitopes, namely AEMQNPVYL, QPRFSNLSM, TPNATSPTL, MPTNTTDITY, and RLTPRPVSR, have been identified as having high conservancy across various strains. Their conservation suggests that they are crucial for the immune recognition process and may play significant roles in eliciting CTL responses. Due to their high degree of conservation, these epitopes are promising candidates for inclusion in future vaccine development, as they are likely to stimulate robust immune responses across a wide range of individuals and populations. The presence of these epitopes in critical viral strains further strengthens their relevance in vaccine design.

Similarly, the MHC class II epitopes NSTNITAVVRAQGLD and CNSTNITAVVRAQGL have shown significant potential due to their conserved nature. These epitopes are essential for activating helper T cells, which are pivotal in promoting both humoral and cellular immunity. Their conservation across various strains suggests that they are vital for maintaining immune responses and may contribute to long-lasting immunity in vaccinated individuals. Both MHC I and MHC II epitopes, due to their high conservancy and immune activation potential, are suggested as key candidates for future vaccine formulations. However, experimental validation is essential to confirm their immunogenicity and effectiveness in eliciting protective immune responses. Further studies, including in vitro assays and in vivo animal models, are required to thoroughly evaluate the ability of these epitopes to induce desired immune responses and assess their potential for broader vaccine applications.

To develop the EBV vaccine construct, the 50S ribosomal protein L7/L12 from Mycobacterium tuberculosis was used as an immunoadjuvant [24,47] and most powerful PRRs of the human innate immune system [48,49]. Several linkers, including GGGS, AAY, GPGPG, and EAAAK, were employed in this study [25,50,51]. Adjuvant connected to the vaccine by EAAAK and CTL/HTL connected by AAY and GPGPG, respectively [52,53]. Moreover, the CTL, HTL, and TAT sequences were separated by GGGS linkers. Analysis of the vaccine showed that it contained a strong antigen, as indicated by antigenic scores from VaxiJen v2.0 and AntigenPro. Good physicochemical properties are crucial for an effective vaccine in terms of formulation, manufacturing, administration, and storage [54]. The ExPASy ProtParam service was used to predict the physicochemical characteristics of the vaccines. The quantity of amino acids, molecular weight, formula, theoretical isoelectric point (pI), total atom count, predicted half-life, instability index, and aliphatic index are some of these properties. The molecular weight of the vaccines was used as a reference for Western blot and SDS-PAGE electrophoresis. The pH at which a molecule has no net electrical charge is shown by the theoretical pI. Furthermore, the vaccine's solubility, toxicity, allergenicity, and transmembrane helices were assessed. The vaccine was discovered to be stable, soluble, non-toxic, non-allergic, and able to mature dendritic cells. TLR4 recognized 50S ribosomal protein L7/L12 as an agonist that can induced the DC maturation that previous study confirmed [21]. Adjuvants are vital components of multi-epitope vaccines as they enhance the immunogenic properties of the vaccines. Moreover, enhancing innate immunity plays a key role in improving the therapeutic effectiveness of vaccine candidates. TLRs are believed to exhibit both hydrophilic and hydrophobic characteristics. Hydrophobic ligands, such as those for TLR1, TLR2, and TLR4, typically bind to internal protein pockets. In contrast, dsRNA, a hydrophilic ligand, interacts with the solvent-exposed surface of TLR3. This interaction plays a crucial role in activating the innate immune response [55]. Overall, the data suggests that the vaccine can stimulate immune responses. The tertiary structure of the constructed vaccines was predicted using AlphaFold [56] and the refinement of the 3D structure was implemented by Galaxyweb [30,57]. The 3D structure was enhanced using Galaxyweb through various assessments including the Ramachandran plot, ERRAT, and Z-score. Molecular docking was performed using the Cluspro tool. The protein-protein interactions within the TLR4-vaccine complex were analyzed, focusing on hydrogen bonds and other interactions. To assess the stability of the complex, MDS were conducted using the GROMACS tool. The RMSD and Rg analyses indicated that the complex was stable. Additionally, the C-ImmSim server was utilized to track the dynamics of the human immune response to the vaccine through in-silico immunological simulations. Following three doses of the vaccine, it was observed that immune responses were significantly enhanced, with a high quantity of antibodies (IgG, IgM) detected. The population coverage showed extensive global reach with 100 % coverage. Our strategy for developing the EBV vaccine integrated immunoinformatics predictions with empirical testing, which involved experimentally validated epitopes. This strategy's primary benefit is that it saves time and money by focusing mostly on proven epitopes rather than hypothesized ones and utilizing immunoinformatics to decide which are better suited for the creation of epitope vaccines [[58], [59], [60]].

## 5 Conclusion

The computational methodologies elucidated in this research endeavor offer the potential to unveil novel insights regarding the development of a multi-epitope vaccine targeting EBV's GP350 protein. Subsequent scrutiny of the newly formulated vaccine necessitates meticulous evaluation through both in-vitro experiments and animal models. Leveraging an immunoinformatics platform, this study delineated a cutting-edge multi-epitope vaccine targeting EBV that exhibits high immunogenicity and possesses the requisite attributes to serve as a carrier vaccine. Integration of epitope-prediction algorithms facilitated the identification and fusion of multiple CTL and HTL epitopes, interconnected by appropriate linkers and adjuvants to augment the immunogenicity of the vaccine. Promising results were obtained from the evaluation of the vaccine's tertiary structure, physiochemical characteristics, antigenicity, and allergenicity. Furthermore, comprehensive analyses involving molecular docking and molecular dynamics simulations were conducted to explore the interaction between the vaccine and TLR4. Subsequent in silico immune simulations validated robust immune cell responses and efficient antigen clearance mechanisms. This investigation harnessed a diverse array of immune-informatics tools to explore various attributes of the vaccine. The evaluation of the subunit vaccine based on predicted epitopes underscores its considerable potential as an immunogenic and viable candidate for combating EBV.

## Funding

Non.

## Declaration of competing interest

The authors declare that they have no known competing financial interests or personal relationships that could have appeared to influence the work reported in this paper.

## Data Availability

The data that has been used is confidential.
